# Effects of Ceramic Shade, Ceramic Thickness, and Surface Treatment on the Color Match of High-Translucency Monolithic Multilayer Zirconia Restorations

**DOI:** 10.1155/ijod/2004922

**Published:** 2025-06-19

**Authors:** Farhad Tabatabaian, Siddharth Vora, Shahriar Mirabbasi

**Affiliations:** ^1^Department of Oral Health Sciences, The University of British Columbia, Vancouver, British Columbia, Canada; ^2^Department of Electrical and Computer Engineering, The University of British Columbia, Vancouver, British Columbia, Canada

**Keywords:** ceramic, color, esthetics, spectrophotometry, zirconia

## Abstract

**Purpose:** The purpose of this in vitro study was to evaluate the effects of ceramic shade, ceramic thickness, and surface treatment on the color match of high-translucency monolithic multilayer zirconia restorations.

**Materials and Methods:** Seventy-two high-translucency monolithic multilayer zirconia disk specimens with different shades (A2, A3, B2, and B3) and different thicknesses (1, 1.5, and 2 mm) were fabricated, polished, and glazed. CIELab values were measured with a spectrophotometer in the incisal, middle, and cervical regions before and after glazing. ∆*E*_00_ color differences were calculated between polished and glazed specimens (Δ*E*_1_), between polished specimens and their analogous Vita classical shade tabs as targets (Δ*E*_2_), and between glazed specimens and the targets (Δ*E*_3_). The ∆*E*_00_ values were compared with a 50:50% acceptability threshold (∆*E*_00_ = 1.8) to assess color matches. Repeated measures ANOVA, Bonferroni, and 1-sample *t*-tests were used for data analysis (*α* = 0.05).

**Results:** Mean ∆*E*_00_ values ranged between 1.11 and 2.74 for Δ*E*_1_, between 2.69 and 6.78 for Δ*E*_2_, and between 1.47 and 5.59 for Δ*E*_3_. The overall mean values were 1.82, 4.66, and 3.63 for Δ*E*_1_, Δ*E*_2_, and Δ*E*_3_, respectively. Ceramic shade, ceramic thickness, and surface treatment significantly affected the CIELab values (*p* < 0.05). All mean ∆*E*_2_ and ∆*E*_3_ values were greater than the threshold (*p* < 0.05) except for the mean ∆*E*_3_ for the 1.5- and 2-mm-thick, A3 shade, glazed zirconia in the cervical region (*p* > 0.05).

**Conclusions:** The color match of high-translucency monolithic multilayer zirconia restorations depends on ceramic shade. Additionally, increased ceramic thicknesses (≥1.5 mm) and glazing can improve the color match of these restorations.

## 1. Introduction

Monolithic multilayer zirconia restorations have provided a unique opportunity to fulfill both mechanical and esthetic requirements in restorative dentistry [1–5]. These restorations need no veneering porcelains, thus eliminating the risk of porcelain chip-off [6, 7], while showing a tooth-like appearance. This is because of their gradation of color and translucency from incisal to cervical regions as opposed to monolithic monolayer zirconia restorations [8, 9]. Additionally, the use of computer-aided design and computer-aided manufacturing (CAD-CAM) systems has simplified the fabrication processes of zirconia restorations [10, 11]. However, achieving color matches for monolithic multilayer zirconia restorations is still challenging because of clinical and laboratory factors affecting the final color of these restorations [12, 13].

Factors such as background, luting agent, ceramic translucency, ceramic thickness, restoration design, and surface treatment can impact the resulting color of monolithic zirconia restorations [14–20]. A discolored or dark background negatively affects the color match of zirconia restorations with decreased thicknesses [21, 22]. The adverse effect of the dark background can be neutralized by using opaque luting agents, raising the ceramic thickness, and applying low-translucency ceramics [23–26]. The restoration design can alter the restoration translucency and thickness, thus affecting the resulting color [16, 27]. A minimum ceramic thickness of 1 mm is recommended to mask the color of underlying backgrounds and luting agents in monolithic zirconia restorations [14, 28–30]. Different surface treatments, such as polishing and glazing, may influence the surface texture, resulting color, and optical properties of ceramic restorations [31, 32]. Glazed monolayer zirconia shows lower lightness and higher yellowness than polished monolayer zirconia [33], and also, glazing reduces the translucency of zirconia, thus improving the ceramic masking ability [34].

There is a common standard method (ISO/TR 28642:2016) in color science in dentistry to evaluate color matches between a restoration and a target based on their CIELab color coordinates (CIELab: *L*^⁣^*∗*^^: lightness, *a*^⁣^*∗*^^: green–red, *b*^⁣^*∗*^^: blue–yellow) [35–37]. The CIELab values are measured with color measuring instruments. The color difference between the restoration and the target is calculated from color difference formulas and compared with visual thresholds [38–40]. A 50:50% acceptability threshold is a color difference that is acceptable to half of the viewers [38, 39]. When the color difference is less than or equal to the threshold, the restoration and the target show a color match, otherwise, they indicate a color mismatch [38–40].

Though the clinical application of a minimum ceramic thickness of 1 mm may ensure proper masking ability of high-translucency monolithic multilayer zirconia restorations [28–30], reports on the influence of ceramic thickness on the color match of these restorations are controversial [14, 41]. Additionally, the impacts of ceramic shade and surface treatment on the color match of high-translucency multilayer zirconia ceramics for monolithic crowns are unclear, and finally, the color match of high-translucency multilayer zirconia with regard to these factors and with reference to Vita classical shade guide as a common clinical shade matching tool is not still fully understood [42, 43]. Therefore, the purpose of this in vitro study was to assess the effects of ceramic shade, ceramic thickness, and surface treatment on the color match of high-translucency monolithic multilayer zirconia restorations. The null hypothesis was that the ceramic shade, ceramic thickness, and surface treatment would not affect the color match of high-translucency monolithic multilayer zirconia restorations.

## 2. Materials and Methods

A total of 72 high-translucency monolithic multilayer zirconia disk specimens with four different shades (A2, A3, B2, and B3) and three different thicknesses (1, 1.5, and 2 mm) were fabricated, polished, and glazed. As such, there were six specimens in each experimental group. The specimens resembled a maxillary right central tooth in terms of shape. The sample size was calculated from a formula $\left(n=\frac{{\left(Z_{1-\frac{\alpha }{2}}|+|Z_{1-\beta }\right)}^{2}\times 2{\text{SD}}^{2}}{d^{2}}\right)$ with *Z* = 1.50, *d* = 1.8, SD = 0.6, *α* = 0.05, and *β* = 0.1 derived from prior relevant studies [14, 16, 23, 28].

**Figure 1:**
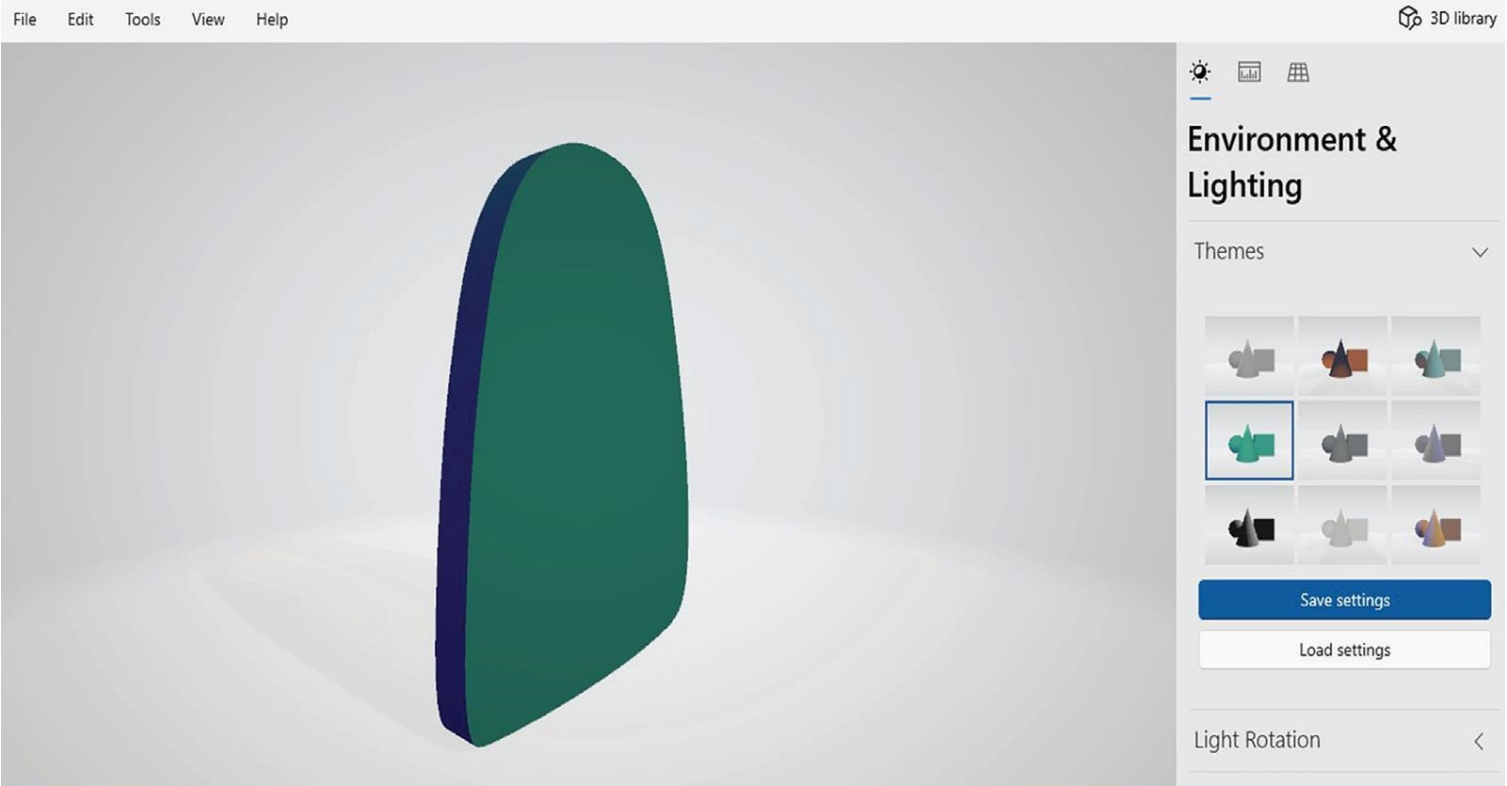
Design of zirconia specimens by using a software program.

The zirconia specimens were designed with software (SOLIDWORKS 2019, Dassault Systèmes, Vélizy-Villacoublay, France) (Figure 1) to have a final width of 8.8 mm and height of 12 mm and nested at the same incisocervical position in 98.5 × 16 mm 4Y-5Y high-translucency multilayer zirconia blanks (Explore Esthetic, Upcera, Shenzhen, China) (Table 1). The specimens were milled with a CAD-CAM system (CORiTEC 250i, imes-icore GmbH, Eiterfeld, Germany) while excluding 1 mm from the top and bottom of the blanks, and then sintered at a maximum temperature of 1480°C for 10 h in a sintering furnace (iSINT HT, imes-icore GmbH, Eiterfeld, Germany) as instructed by the manufacturer. A digital gauge (293 MDC-MX Lite, Mitutoyo Corp, Tokyo, Japan) was used repeatedly to ensure an accurate zirconia thickness (±0.02 mm). Specimens outside the mentioned thicknesses were replaced. The external surface of the veneer was polished by an expert dental technician with a polishing kit (BruxZir, Glidewell Direct, Irvine, CA, USA) for 5 min in a two-step process including a green cup using light pressure and no water and an orange cup using light-to-medium pressure with a single directed motion and no water according to the manufacturer's instruction. All zirconia specimens were cleaned in a bath containing 98% ethanol for 20 min, rinsed with water, and air-dried.

**Figure 2:**
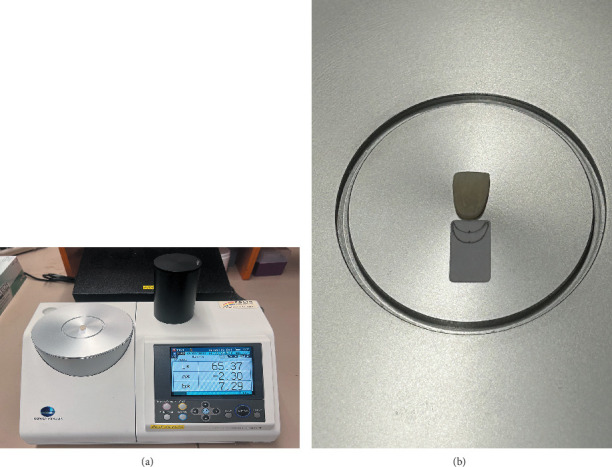
(A) Color measurement for the specimen and (B) the customized scaled label for the region specification.

The CIELab color coordinates of the specimens' external surface were measured with a benchtop laboratory spectrophotometer (CM-5 Spectrophotometer, Konica Minolta, Tokyo, Japan) by a skilled operator in three tooth regions: incisal, middle, and cervical (Figure 2A). The device had a diffuse illumination integrating sphere system (d/8: diffuse illumination, 8° viewing), a wavelength range of 360–740 nm, a wavelength pitch of 10 nm, a pulsed xenon light source, and a precision of ∆*E*_ab_ = 0.04. The device was set at reflectance mode, SCE (specular component excluded), 3-mm measurement area (aperture), a 2° standard observer, D65 illuminant, three-time repeated measurements, and CIELab colorimetric data [44]. To stabilize the location of the specimens on the measuring port of the device and to specify the specimens' incisal, middle, and cervical regions throughout color measurements, a customized scaled label was adhered onto a target mask (Figure 2B). A zero-calibration black cylindric box was placed on the specimens to isolate them from external light during color measurements. The device was automatically calibrated with its internal white plate. The average CIELab values for each specimen were recorded.

The external surface of the specimens was glazed with a thin layer of clear liquid (DD contrast glaze clear; Dental Direkt GmbH, Spenge, Germany) in a furnace (Programat EP 3010 Porcelain Furnace, Ivoclar Vivadent, Schaan, Liechtenstein) with a heating rate of 50°C/min while temperature was rising from 400°C to 830°C. Then, the color measurements were repeated for the specimens' external surface with the same device by the same operator under the same conditions and settings. Analogous Vita classical shade tabs for the specimens (A2, A3, B2, and B3) were selected from a new intact shade guide (VITA classical A1–D4 shade guide, VITA Zahnfabrik H. Rauter GmbH and Co. KG, Bad Säckingen, Germany) and considered as targets. The CIELab values of the labial surface of the shade tabs were measured on their incisal, middle, and cervical regions with the same device by the same operator under the same conditions and settings (Table 2).

Δ*E*_00_ color difference values were calculated from the formula:

  $$\begin{array}{c} \text{CIEDE}2000=\Delta E_{00}=\sqrt{{\left(\frac{\Delta L^{\prime }}{k_{L}S_{L}}\right)}^{2}+{\left(\frac{\Delta C^{\prime }}{k_{C}S_{C}}\right)}^{2}+{\left(\frac{\Delta H^{\prime }}{k_{H}S_{H}}\right)}^{2}+R_{T}\times \left(\frac{\Delta C^{\prime }}{k_{C}S_{C}}\right)\times \left(\frac{\Delta H^{\prime }}{k_{H}S_{H}}\right)}, \end{array}$$

with *k*_*L*_ = 2, *k*_*C*_ = 1, and *k*_*H*_ = 1 [38–40]. The Δ*E*_00_ color differences were calculated for each specimen between after polishing and after glazing (Δ*E*_1_) to assess the color differences induced by surface treatments of polishing and glazing, and also between each specimen and its analogous Vita classical shade tab after polishing (∆*E*_2_) and after glazing (∆*E*_3_) to evaluate the color matches between specimens and the targets (Table 2). An acceptability threshold (Δ*E*_00_ = 1.8) was assumed to evaluate if the ∆*E*_1_, ∆*E*_2_, and ∆*E*_3_ color differences were clinically acceptable [38, 39]. A color match could be achieved when the color difference value was ≤1.8.

Data were statistically analyzed with software (IBM SPSS Statistics v26, IBM Corp, New York, NY, USA). The Kolmogorov–Smirnov test showed a normal distribution of data (*p* > 0.05). Repeated measures ANOVA was used to assess the effects of ceramic shade, ceramic thickness, and surface treatment on the ∆*E*_1_, ∆*E*_2_, and ∆*E*_3_ for the three tooth regions separately. Pairwise comparisons of different combinations of the ceramic shade, ceramic thickness, and surface treatment were performed with the Bonferroni test for the three tooth regions separately, with specific orders considering ceramic shade, ceramic thickness, and surface treatment. With software (STATA, StataCorp LP, Lakeway, TX, USA), a 1-sample *t*-test was used to compare the Δ*E*_1_, Δ*E*_2_, and Δ*E*_3_ values of the studied combinations with the threshold (Δ*E*_00_ = 1.8) (*α* = 0.05 for all tests).

## 3. Results

**Figure 3:**
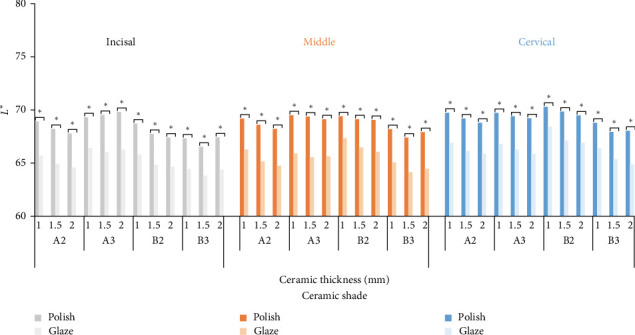
Mean *L*^⁣^*∗*^^ (lightness) values (*⁣*^*∗*^ represents significant differences with *p* < 0.05).

**Figure 4:**
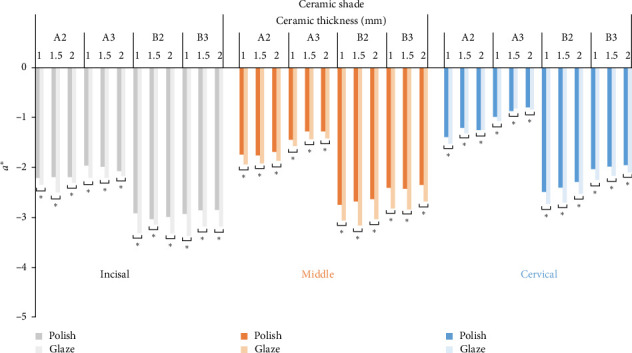
Mean *a*^⁣^*∗*^^ (green–red) values (*⁣*^*∗*^ represents significant differences with *p* < 0.05).

**Figure 5:**
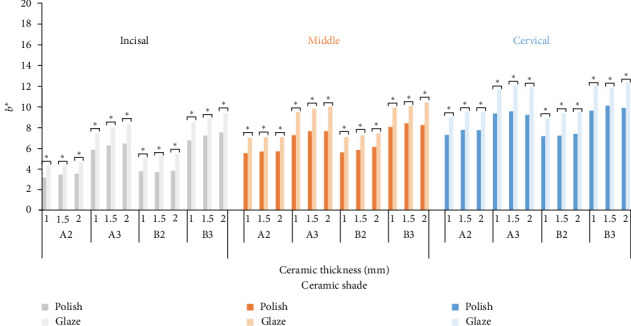
Mean *b*^⁣^*∗*^^ (blue–yellow) values (*⁣*^*∗*^ represents significant differences with *p* < 0.05).

**Figure 6:**
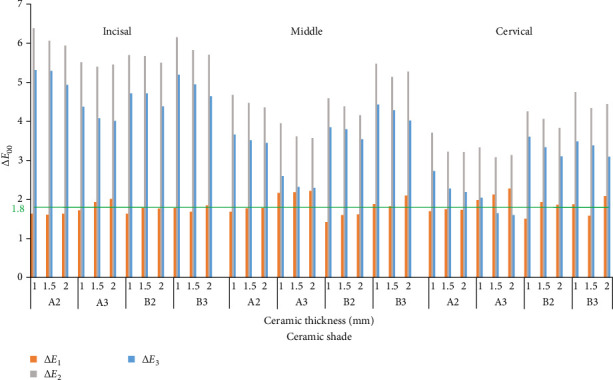
Mean Δ*E*_1_ (color difference between polished and glazed zirconia), Δ*E*_2_ (color difference between polished zirconia and its analogous Vita classical shade tab), and Δ*E*_3_ (color difference between glazed zirconia and its analogous Vita classical shade tab) values with reference to the acceptability threshold of 1.8 (green line).

The means of the CIELab, Δ*E*_1_, Δ*E*_2_, and Δ*E*_3_ values for different combinations of the ceramic shade (A2, A3, B2, B3), ceramic thickness (1, 1.5, 2 mm), surface treatment (Polish, Glaze), and tooth region (Incisal, Middle, Cervical) are presented in Figures 3–6. The ANOVA with between-subjects factors (shade and thickness) showed that the shade, thickness, and their interaction affected the Δ*E*_1_, Δ*E*_2_, and Δ*E*_3_ values (*p* < 0.001) (Table 3). Pairwise comparisons of the CIELab values for the diverse combinations of shade, thickness, and surface treatment indicated significant differences between various shades/thicknesses with the same surface treatment and between various surface treatments with the same shade/thickness (*p* < 0.05). Also, pairwise comparisons of the Δ*E*_1_, Δ*E*_2_, and Δ*E*_3_ values for the diverse combinations of shade and thickness revealed significant differences between various shades with the same thickness and between various thicknesses with the same shade (*p* < 0.05).

Results of the 1-sample *t*-test for evaluation of the Δ*E*_1_ (color differences between polished and glazed specimens), Δ*E*_2_ (the color match of polished specimens), and Δ*E*_3_ (the color match of glazed specimens) revealed that some tested combinations had ∆*E*_1_ values beyond the acceptability threshold of 1.8 (*p* < 0.05) (Figure 6). Also, all tested combinations had the ∆*E*_2_ and Δ*E*_3_ values beyond the acceptability threshold of 1.8 (*p* < 0.05) except for the combinations of A3-1.5 mm-Glaze-Cervical and A3-2 mm-Glaze-Cervical (*p* > 0.05) (Figure 6).

## 4. Discussion

Ceramic shade, ceramic thickness, and surface treatment significantly affected the color match of high-translucency monolithic multilayer zirconia restorations based on the results. Thus, the null hypothesis of the study was rejected.

The color difference between polished and glazed zirconia was evaluated by measuring the Δ*E*_1_ values, with the overall mean Δ*E*_1_ value of 1.82. Accordingly, some color differences between polished and glazed zirconia were greater than the threshold (Δ*E*_1_ >1.8), regardless of the ceramic shade, thickness, and region (Figure 6), resulting in unacceptable color mismatches between polished and glazed zirconia due to the significant changes in *L*^⁣^*∗*^^ and *b*^⁣^*∗*^^ values (Figures 3 and 5).

As per results, glazing after polishing extremely diminished the *L*^⁣^*∗*^^ value, raised the *b*^⁣^*∗*^^ value, and slightly reduced the *a*^⁣^*∗*^^ value (Figures 3, 4, and 5). The *L*^⁣^*∗*^^ value depends on the amount of reflected light from the zirconia surface as well as the light scattering of the zirconia surface. The more the amount of reflected light and light scattering of the zirconia surface, the more the *L*^⁣^*∗*^^ value would be [31, 33]. Glazing raises the spectral reflectance by creating a smooth, glossy surface; however, a glazed zirconia surface mainly reduces light scattering [33]. This may be a reason for the *L*^⁣^*∗*^^ reduction. Moreover, though a glazed glossy surface maximizes the ratio of the specular to diffuse component of the reflected light [44], the SCE mode set for the spectrophotometer used in the current study excluded the specular component of the spectral reflectance. This may be an explanation for the decrease in the *L*^⁣^*∗*^^ value. The SCE, rather than the specular component included (SCI) mode, was used in the present study, as the SCE mode better approximates the view of the human eye based on an accepted theory in color science [44]. The increase of the *b*^⁣^*∗*^^ value and the decrease of the *a*^⁣^*∗*^^ value may be linked to additional firing of glazing. Additional firing changes zirconia structure and metal oxides, leading to an increase in the yellowness and a decrease in the redness [31–33]. The metal oxides derangement, which seems to be shade sensitive due to the zirconia metal oxide content, may address color changes after glazing [31–33].

This result is in agreement with Kim et al. [33] but in disagreement with Manziuc et al. [34]. This may be due to the same optical geometry of the benchtop spectrophotometers used in the former study and the current investigation (diffuse illumination/8° viewing) as opposed to the latter study, in which a handheld spectrophotometer with a different optical geometry was employed.

The color match between several high-translucency multilayer zirconia shades and their analogous Vita classical shades (the targets) was assessed by measuring the Δ*E*_2_ (for polished zirconia) and Δ*E*_3_ (for glazed zirconia) values. Accordingly, the color differences were less than the threshold (≤1.8) only for 1.5- and 2-mm-thick, A3 shade, glazed multilayer zirconia in the cervical region (*p* > 0.05 for the 1-sample *t*-test) (Figure 6), resulting in acceptable color matches. This shows that color matches in high-translucency monolithic multilayer zirconia restorations depend on the restoration's shade, thickness, surface treatment, and region. Though the color match of glazed zirconia (Δ*E*_3_) depends on the restoration's shade and thickness, it is not sensitive to their interaction (Table 3). This means that the effect of shade on the color match doesn't depend on the amount of thickness and vise versa, as a result, these two factors should be clinically considered and controlled separately.

In addition, color matches seem to be sensitive to the restoration's shade and region, as acceptable color matches were only gained for A3 shade zirconia in the cervical region (Figure 6). This may be attributed to the manufacturing process of multilayer zirconia, which determines the type, distribution, and density of coloring metal oxides in different layers of this ceramic [3–5, 8, 13]. The cervical region of multilayer zirconia showed the lowest color difference values among all regions, regardless of the shade (Figure 6), as multilayer zirconia is more chromatic and less translucent in its cervical region due to the highest density of metal oxides of this ceramic region [12]. This enhances the ceramic masking ability and subsequently the possibility of color matching [13].

However, the same shades of high-translucency multilayer zirconia and Vita classical tabs did not mostly match. This color mismatch may be due to the difference in their type of ceramic. Yttria-stabilized tetragonal zirconia polycrystals differ from Vita classical handmade feldspathic ceramic shade tabs in terms of structure and optical properties [9, 12]. Furthermore, zirconia restorations and Vita classical shade tabs did not have the same thickness, thus leading to color mismatches. This is consistent with the reports that prioritize the use of the custom shade guides made of original ceramics rather than universal shade guides, which can be a source of bias for color matching, thus emphasizing the use of custom shade guides for higher accuracies [42, 43, 45, 46].

According to the results, as the ceramic thickness increased, the color difference decreased, and therefore, higher zirconia thicknesses (≥1.5 mm) created color matches as opposed to lower zirconia thicknesses (1 mm). Comparably, the need for a sufficient ceramic thickness for achieving color matches has been emphasized in the literature because of masking the backgrounds and optimizing the optical properties and color of ceramic restorations [12, 14, 16, 18, 21, 23]. Hence, in spite of the fact that 1-mm-thick multilayer zirconia seems durable mechanically [47], increased thicknesses (≥1.5 mm) ensure esthetics based on the current study result. However, this result is not confirmed by a study [13] that suggested using 1-mm-thick high-translucency multilayer zirconia for esthetics. This may be due to the use of a background with a shade similar to the target in that study [13], thus compensating for the decreased zirconia thickness tested, resulting in color matches.

As shown in Figure 6, ΔE_3_ mean values (glazing) were less than their corresponding ΔE_2_ mean values (polishing) in all experimental groups, regardless of the ceramic shade, thickness, and region. Thus, glazing outperformed polishing by reducing color mismatches (Figure 6). This may be due to the fact that glazing better reproduces the optical characteristics of the glossy surface of the shade tabs and human teeth. Furthermore, glazing moderates the zirconia lightness by reducing light scattering while improving the zirconia yellowness by modifying the zirconia metal oxides [31, 33, 34].

Different color difference formulas have been used in dentistry; however, the CIEDE2000 formula is the most accurate one because of offering accurate weights for color coordinates [35]. Hence, the CIEDE2000 was used in this study with consideration of *k*_*L*_ = 2, *k*_*C*_ = 1, and *k*_*H*_ = 1, as the CIEDE2000 (2:1:1) formula outweighs CIEDE2000 (1:1:1) for dental ceramics [40]. Also, considering a visual threshold is essential for the interpretation of color differences and the evaluation of color matches [35, 37, 38]. Accordingly, the 50:50% acceptability threshold of ∆*E*_00_ = 1.8, which was determined by 175 observers with diverse experiences in a multicenter investigation [39], was assumed in the present study.

Based on the current study results, only accurate shade selection using a universal shade guide does not ensure optimum color matching results. Specifically, other factors such as ceramic thickness and surface treatment also play crucial roles. Hence, high-translucency monolithic multilayer zirconia crowns should be fabricated with a minimum thickness of 1.5 mm and glazed rather than just polished to achieve better color matching. Also, color matching of these crowns is also dependent on ceramic shade. These need to be concerned by dental clinicians, technicians, and manufacturers. Backgrounds and cements may impact the color match of zirconia restorations; however, their impacts were not assessed in this study. Moreover, though using the flat specimens must have minimized the minor inaccuracies of small-aperture spectrophotometry due to edge loss effects of curved surfaces [35], it might not reflect the clinical setting of real crowns with curvatures. Finally, only one brand of multilayer zirconia with a specific translucency and in four shades was assessed with reference to the Vita classical shade guide. These are the limitations of the current study. Hence, testing different backgrounds and cements and various multilayer zirconia crowns/brands/translucencies/shades with reference to custom zirconia shade guides is suggested for future research.

## 5. Conclusions

Within the limitations of this study, the subsequent conclusions were drawn:

1. Ceramic shade, ceramic thickness, and surface treatment affected the color match of high-translucency monolithic multilayer zirconia restorations.
2. The color match of high-translucency monolithic multilayer zirconia restorations is sensitive to ceramic shade.
3. Increased ceramic thicknesses (≥1.5 mm) and glazing can improve the color match of these restorations.

## Figures and Tables

**Table 1:** Features of the zirconia ceramic used.

Flexural strength	727–1000 MPa	

Translucency	47%–48.8%	

Physical characteristics	Density after sintering	≥6.0 g (cm)^3^
	CTE (25–500°C)	(10.5 ± 0.5) × 10^−6^K^−1^
	Chemical solubility after sintering	<100 μg/cm^2^
	Radioactivity	<0.1 Bq/g
	Sintering temperature	1480°C

Chemical composition	ZrO_2_ + HfO_2_ + Y_2_O_3_	>96.5%
	Y_2_O_3_	5.8%–9.7%
	Al_2_O_3_	<0.5%
	Fe_2_0_3_	<0.5%
	Er_2_O	<2.0%
	Others oxides	<0.5%

**Table 2:** CIELab values of vita classical shade tabs (targets).

Shade tab	Section	*L* ^⁣^*∗*^^	*a* ^⁣^*∗*^^	*b* ^⁣^*∗*^^
A2	Incisal	62.32	−0.08	9.20
	Middle	64.31	0.10	9.81
	Cervical	66.08	0.16	10.91

A3	Incisal	60.72	0.04	9.90
	Middle	63.92	0.17	10.43
	Cervical	64.67	0.29	12.21

B2	Incisal	62.65	−1.20	9.31
	Middle	64.76	−0.83	9.66
	Cervical	66.12	−0.28	10.30

B3	Incisal	60.00	−0.41	12.10
	Middle	61.75	−0.22	13.26
	Cervical	64.30	−0.15	14.97

**Table 3:** Results of repeated measures ANOVA for effects of ceramic shade and ceramic thickness on color differences in different tooth regions.

Tooth region	Color difference	Source (between-subjects effects)	Type III sum of squares	Mean square	*F*	*p*
Incisal	Δ*E*_1_	Shade	0.652	0.217	4.502	**0.006**
		Thickness	0.183	0.091	1.894	0.159
		Shade × thickness	0.277	0.046	0.957	0.462
	Δ*E*_2_	Shade	4.770	1.590	61.312	**<0.001**
		Thickness	0.992	0.496	19.120	**<0.001**
		Shade × thickness	0.414	0.69	2.661	**0.023**
	Δ*E*_3_	Shade	10.511	3.504	156.402	**<0.001**
		Thickness	2.022	1.011	45.138	**<0.001**
		Shade × thickness	0.302	0.050	2.250	0.050

Middle	Δ*E*_1_	Shade	4.047	1.349	23.631	**<0.001**
		Thickness	0.230	0.115	2.016	0.142
		Shade × thickness	0.185	0.031	0.541	0.775
	Δ*E*_2_	Shade	22.820	7.607	468.253	**<0.001**
		Thickness	1.499	0.750	46.146	**<0.001**
		Shade × thickness	0.229	0.038	2.346	**0.042**
	Δ*E*_3_	Shade	32.403	10.801	627.063	**<0.001**
		Thickness	1.184	0.592	43.356	**<0.001**
		Shade × thickness	0.188	0.031	1.823	0.110

Cervical	Δ*E*_1_	Shade	1.777	0.592	7.725	**<0.001**
		Thickness	0.621	0.310	4.048	**0.022**
		Shade × thickness	1.017	0.170	2.212	0.054
	Δ*E*_2_	Shade	20.347	6.782	252.910	**<0.001**
		Thickness	1.972	0.986	36.758	**<0.001**
		Shade × thickness	0.357	0.059	2.217	0.054
	Δ*E*_3_	Shade	32.147	10.716	493.433	**<0.001**
		Thickness	2.710	1.355	62.384	**<0.001**
		Shade × thickness	0.265	0.044	2.034	0.075

*Note:* Δ*E*_1_: color difference between polished and glazed zirconia, Δ*E*_2_: color difference between polished zirconia and its analogous vita classical shade tab, and Δ*E*_3_: color difference between glazed zirconia and its analogous vita classical shade tab (bold values show *p* values of <0.05).

## Data Availability

The data that support the findings of this study are available from the corresponding author upon reasonable request.
